# Effects of Different Types of Cognitive Training on Cognitive Function, Brain Structure, and Driving Safety in Senior Daily Drivers: A Pilot Study

**DOI:** 10.1155/2015/525901

**Published:** 2015-06-16

**Authors:** Takayuki Nozawa, Yasuyuki Taki, Akitake Kanno, Yoritaka Akimoto, Mizuki Ihara, Ryoichi Yokoyama, Yuka Kotozaki, Rui Nouchi, Atsushi Sekiguchi, Hikaru Takeuchi, Carlos Makoto Miyauchi, Takeshi Ogawa, Takakuni Goto, Takashi Sunda, Toshiyuki Shimizu, Eiji Tozuka, Satoru Hirose, Tatsuyoshi Nanbu, Ryuta Kawashima

**Affiliations:** ^1^Smart Ageing International Research Center, Institute of Development, Aging and Cancer, Tohoku University, Sendai 980-8575, Japan; ^2^Division of Developmental Cognitive Neuroscience, Institute of Development, Aging and Cancer, Tohoku University, Sendai 980-8575, Japan; ^3^Division of Medical Neuroimage Analysis, Department of Community Medical Supports, Tohoku Medical Megabank Organization, Tohoku University, Sendai 980-8575, Japan; ^4^Department of Functional Brain Imaging, Institute of Development, Aging and Cancer, Tohoku University, Sendai 980-8575, Japan; ^5^Japan Society for the Promotion of Science, Tokyo 102-8472, Japan; ^6^Research Division 2, Mobility Services Laboratory, Nissan Motor Co., Ltd., Kanagawa 243-0123, Japan; ^7^CAE and Testing Division 1, Vehicle Test and Measurement Technology Development, Nissan Motor Co., Ltd., Kanagawa 243-0192, Japan; ^8^Research Division 2, Prototype and Test Department, Nissan Motor Co., Ltd., Kanagawa 243-0123, Japan

## Abstract

*Background*. Increasing proportion of the elderly in the driving population raises the importance of assuring their safety. We explored the effects of three different types of cognitive training on the cognitive function, brain structure, and driving safety of the elderly. *Methods*. Thirty-seven healthy elderly daily drivers were randomly assigned to one of three training groups: Group V trained in a vehicle with a newly developed onboard cognitive training program, Group P trained with a similar program but on a personal computer, and Group C trained to solve a crossword puzzle. Before and after the 8-week training period, they underwent neuropsychological tests, structural brain magnetic resonance imaging, and driving safety tests. *Results*. For cognitive function, only Group V showed significant improvements in processing speed and working memory. For driving safety, Group V showed significant improvements both in the driving aptitude test and in the on-road evaluations. Group P showed no significant improvements in either test, and Group C showed significant improvements in the driving aptitude but not in the on-road evaluations. *Conclusion*. The results support the effectiveness of the onboard training program in enhancing the elderly's abilities to drive safely and the potential advantages of a multimodal training approach.

## 1. Introduction

The proportion of the elderly in the driving population is increasing rapidly, especially in advanced countries, and assuring their safety as drivers is an issue of huge importance [1, 2]. Promotion of age-based license reassessments and the recommendation of driving cessation are measures undertaken in a number of countries. However, in widespread suburban areas, a private car is a necessary transportation method and driving cessation can result in a large negative impact on quality of life [3]. Thus, methods that enhance driving safety and prolong the mobility of elderly people are important.

Previous studies have indicated that cognitive function has a significant effect on the driving safety of elderly people, both in healthy people and those with mild cognitive impairment [4–6] (however, also see [7]). By focusing on this association between cognitive function and driving safety, many studies have introduced different types of cognitive training and investigated their transfer effects on driving safety and the maintenance of mobility [8–13].

With respect to the general outcomes of cognitive and behavioral training in the elderly, studies have shown the effects of such training interventions on neural structure [14–19] and function [20, 21] in the elderly. However, to the best of the authors' knowledge, there have been no studies specifically addressing the possible association among cognitive enhancement, neural plasticity, and changes in driving safety. Simultaneously investigating the changes in these aspects can help attain a deeper understanding of how the mechanisms of cognitive training can result in changes in behaviors as complicated as driving. Moreover, by comparing the effects of different types of training on cognitive function, brain structure, and driving safety, one can expect to obtain insight into how to design a more effective training program for the optimal driving safety of the elderly. Therefore, we conducted a pilot intervention study and investigated the effects of three different types of cognitive training on cognitive function, brain structure, and the driving safety of the elderly.

Our first aim was to verify the effectiveness of an onboard cognitive training system that we devised to support the elderly in attaining and maintaining their ability to drive safely. The system was designed to enhance processing speed, executive control, working memory, visual processing, and divided attention, which have all been suggested to have significant relevance to driving safety [4, 6, 22]. It provided two types of training tasks (with different levels of working memory demands), which required the users in the driver's seat to respond to light stimuli presented in their peripheral visual field with the steering wheel and brake pedal, according to a set of given rules. More detailed description of the system is provided in Materials and Methods. The system was implemented onboard with the aim of providing elderly drivers with casual and effective cognitive training on a daily basis. To explore the possible positive effects of setting up an onboard cognitive training system, we included a training group that had cognitive training on a personal computer (PC) with a training program that was similar to that used in the onboard cognitive training but that differed in several driving-related factors, such as the required field of view and the motor control involving distributed parts of the body.

Another aim of the study was to investigate the effects of training in a daily intellectual activity, such as puzzle solving, on driving safety. A considerable number of studies have indicated the beneficial effects of such activities in the enhancement or maintenance of cognitive functions in the healthy elderly [23–26]. However, inconsistencies and debates on the effectiveness exist [27–29], and the effects of training in cognitively engaging daily activities are still unclear. In particular, their effects on driving safety have not been directly addressed. Therefore, in this study, we included a crossword-puzzle training group as another cognitive training group, and this type of cognitive training had less apparent relevance to driving safety.

Thirty-seven healthy elderly (age range, 60–75 years) subjects were randomly assigned to one of three training groups: Group V trained in a vehicle with onboard cognitive training, Group P trained with cognitive training on a PC, and Group C trained in solving crossword puzzles. These groups had 24 sessions of training, with each session taking 20 min, over a period of 8 weeks. Before and after the training period (Pre and Post), they underwent tests on cognitive function, brain structure, and driving safety. The changes in cognitive functions induced by the different types of training were evaluated by a neuropsychological test battery that covered a broad range of cognitive functions. The changes in brain structure were investigated using voxel-based morphometry (VBM) analysis of regional gray matter volume (rGMV) and a tract-based spatial statistics (TBSS) analysis of white matter integrity. VBM is a method that is widely used to investigate regional gray matter structural changes induced by training interventions [15, 18, 19, 30–33]. Fractional anisotropy (FA) maps obtained from diffusion tensor imaging (DTI) give indicators of white matter fiber structural integrity, and its changes have also been used to characterize brain structural changes that are induced by training interventions [15, 34–38]. TBSS is a new method which aimed to alleviate the problems of cross-subject FA-map alignment [39]. Driving safety was evaluated by professional driving instructors while the subjects were actually driving on the course of a driving school as well as with a driving aptitude test unit.

## 2. Materials and Methods

### 2.1. Ethics Statement

This study was approved by the Ethics Committee of the Tohoku University Graduate School of Medicine. Written informed consent was obtained from each subject. The study was conducted according to the principles expressed in the Declaration of Helsinki.

### 2.2. Randomized Controlled Trial Design

**Figure 1:**
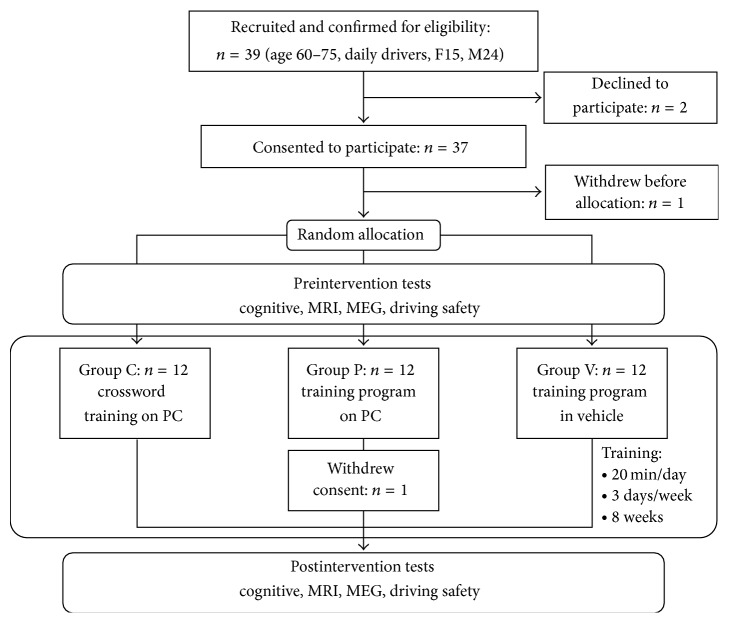
Flow diagram of this study.

Although being a pilot study, this study was registered in the UMIN Clinical Trial Registry (UMIN000006268). The study was conducted between July 2011 and December 2011 in Sendai City, Miyagi Prefecture, Japan. The flow diagram of this study is shown in Figure 1. The protocol is approved by the Ethics Committee.

The study was a double-blind intervention. The subjects and testers were blinded to the study's hypothesis. The subjects were only informed that the study was designed to investigate the effects of three different training programs, and they were blinded to the training of the other two groups. The testers were blinded to the group membership of the subjects. A researcher (T. N.) randomly assigned the subjects who were stratified by sex to one of the three groups by a random draw with a computer (see below for details).

### 2.3. Subjects

The subjects were recruited from local residents through advertisements in a local town paper. The interested applicants were screened firstly by a semistructured telephone interview and then using a questionnaire. Thirty-nine eligible applicants were invited to Tohoku University for a briefing and two people declined to participate after being explained about the study. All consented subjects (*n* = 37) were right-handed, and they were native Japanese speakers. They were aged within the range of 60–75 years at the time of participation, and they were daily drivers who owned a car and who drove more than three times a week on an average. The subjects were not using any medications known to interfere with cognitive function, including benzodiazepines, antidepressants, or other central nervous agents. They had no history of head trauma, mental disease, or diseases known to affect the central nervous system, including thyroid disease, multiple sclerosis, Parkinson's disease, stroke, severe hypertension, or diabetes. To exclude those subjects with potential dementia, the exclusion criterion of Mini-Mental State Examination (MMSE) scores less than 25 was applied [40]. None of the subjects were excluded on the basis of this criterion.

All the subjects provided informed consent, which was approved by the Ethics Committee of the Tohoku University Graduate School of Medicine, in order to participate in this study. After informed consent was obtained, the subjects were randomly assigned to one of the three groups (Group V, P, or C, explained below). Male and female elderly drivers were expected to show distinct driving behaviors [41]. To counterbalance the effect of such baseline difference, stratified randomization was performed [42]. Subjects were blocked into separate strata by their sexes and allocated separately into the three groups by a random draw from a computerized random number generator, achieving a balanced sex ratio among the groups. One subject withdrew consent before the group allocation, and one subject allocated to group P dropped out during the intervention period, resulting in a final total of 35 subjects who were included in the analyses (Figure 1).  Table 1 summarizes the baseline demographics and neuropsychological characteristics of the subjects. We observed no significant differences in any of the characteristics among the groups.

### 2.4. Overview of the Interventions

The subjects were randomly assigned to one of the three intervention groups, Group V, P, or C, which involved a specific type of training program: Group V trained in a vehicle with an onboard system that we developed, Group P trained with a similar program but on a PC, and Group C trained to solve crossword puzzles. Subjects visited our laboratory 24 times (3 days a week for 8 weeks) and they performed 20 min of training each time. All the training was conducted in the presence of time keepers (part-time university students) who confirmed proper task execution by the subjects and provided the necessary help with regard to operating the training programs. Before and after the training intervention period, the subjects underwent neuropsychological and behavioral tests, structural brain imaging [magnetic resonance imaging (MRI)], functional brain measurement by magnetoencephalography (MEG) which will not be described in this paper, and two types of driving safety tests. The primary outcome measure was the driving aptitude test. The preintervention (Pre) tests were conducted within a week before the start of each subject's intervention period, and the postintervention (Post) tests were conducted within a week after the end of the intervention period. In the following sections, the training and Pre/Post tests are explained in more detail.

### 2.5. Training for Each Group

#### 2.5.1. Group V: In-Vehicle Cognitive Training

*Onboard Cognitive Training System*. We developed a cognitive training system that, once installed, can be utilized easily and constantly by elderly daily drivers. Considering their relative tendency to be less familiar with and/or enthusiastic about using computers and video games, we expected that the elderly people would engage in a cognitive training more easily and continuously if it was implemented onboard for use during the frequent occasions of daily driving (e.g., before leaving/after coming home, or in the parking lot of a shopping mall). It was also expected that the training program situated onboard, entailing the use of the visual field common to driving and operable with the familiar steering wheel and brake pedal, would facilitate the transfer of training effects to actual driving situations. To ensure safety, the system was intended to be used while the car was parked.

The system consisted of five light-emitting diodes (LEDs), a speaker, a controller area network interface, and a mobile computer. The LED lights were placed around the driver's seat in the peripheral visual field of the driver. Four lights (top-right, bottom-right, top-left, and bottom-left) were located approximately equidistant from the center of the driver's view, and one light was beside the left (opposite to the driver's seat) side mirror. The location beside the side mirror was chosen considering the importance of paying attention to the side mirror for driving safety. In the training task, the LEDs rhythmically and randomly presented a pattern of lights with various colors. The users responded with the steering wheel and brake pedal according to a set of given rules. The accelerator pedal was not used. Audio feedback informed the subjects of the accuracy of their responses.

The system provided the following two types of training tasks: an immediate response task and a delayed response task. The immediate response task focused on the functions of processing speed, executive control, visual processing, and divided attention. The delayed response task focused on those listed above and working memory. The rules of the two tasks are explained below.

*Immediate Response Task*. The users responded to the rhythmically presented light stimuli on the four LEDs located around the center with the steering wheel. When two lights of the same color (green or blue) were presented on the same side (left or right), the subjects were to turn the wheel in the opposite direction as if to avoid an obstacle on that side. Yellow lights were distractors, and thus the subjects were to suppress their response even if two yellow lights were presented on the same side. In addition, red lights were presented randomly on any of the five LEDs, sometimes in synchronization with the rhythm and sometimes out of the rhythm. The users were to respond to the red light regardless of its position. In particular, when two lights of the same color (green or blue) were on the same side and a red light was on one of the remaining LEDs, they were required to conduct both steering and braking responses. Both the steering wheel and brake were to be operated when the corresponding stimuli were shown, and they were to be returned to the neutral position thereafter.

*Delayed Response Task*. In this task, the light stimuli were presented in the same way as in the immediate response task. The difference was that the users were to delay their steering responses to the periodic stimuli by *n* ( = 1,2,…) steps. Therefore, the users were required to simultaneously judge the current light pattern, memorize the operation in order to conduct its *n* steps later, and conduct a wheel operation in response to *n*-steps-back stimuli if necessary. The load to working memory increased as larger *n* was used. In the present study, we used *n* = 1 for the first 12 visits and *n* = 2 for the later 12 visits. In contrast, brake responses to the random red lights were to be given without delay, as in the immediate response task. In particular, the users were to conduct both steering and braking responses when two lights of the same color (green or blue) were on the same side *n* step(s) before and a red light was on one of the remaining LEDs.

*Adaptive Training with Dynamically Adjusted Tempo*. In both tasks, three successive correct responses induced a change in the task to a tempo in which the rhythmic stimuli were one level faster, and two successive wrong responses induced a change to a level that had a slower tempo. There were 10 levels for tempo, and Table 2 shows the period of the rhythmic stimuli for each level. The dynamically adjusted tempo made the task involve adaptive training, in which the difficulty was always adequately challenging despite the users' changing learning levels. In addition, the training system provided three types of level-dependent background music, with changes between levels 3 and 4 and between levels 6 and 7.

*Training Procedure*. The onboard system was equipped onto a passenger car placed in the parking lot of our institute, and the subjects in Group V individually came and performed two sessions of the immediate response task and two sessions of the delayed response task on each training day. Each session took 5 min. For the delayed response task, the required delay for the steering response to the periodic stimuli was set to *n* = 1 for the first 12 training days and to *n* = 2 for the later 12 training days. At the end of each training session, the system provided a visual feedback that informed the subject of the transition of performance.

#### 2.5.2. Group P: On-PC Cognitive Training

*On-PC Training Tasks*. As a counterpart of the onboard training system, we also developed an on-PC cognitive training system. This system also provided the two types of tasks, the immediate response and delayed response tasks, with the same rules as those explained above. The main differences were as follows. (1) This system presented colored stimuli on a PC display instead of LED lights. The four LEDs around the center of the users' visual field were replaced by four corners of a 24-inch display. The visual angle from the center to the four stimuli positions was 20°. The fifth distant LED beside the side mirror was replaced by stimuli presentation at the center position. (2) Instead of a steering wheel and brake pedal, all responses were made with a three-button computer mouse. Steering to the left and right was replaced by clicking the left button with the right index finger and the right button with the right ring finger, respectively. The brake pedal was replaced by clicking the middle button with the right middle finger.

Because of the shared features of the tasks, we assumed that the basic demands on the functions of processing speed, executive control, and working memory would not be different from that of the onboard cognitive training. However, we also expected that because of the comparatively narrower visuospatial and modality distributions in the input stimuli (limited use of peripheral visual field) and the output effectors (only right-hand fingers) than those of the onboard training, the effects on the functions of visual processing and divided attention would be smaller. In addition, the differences in the similarities to actual driving were expected to make the transfer of this on-PC training to driving safety less likely than that of the onboard training.

*Training Procedure*. Each subject in Group P visited a room in our institute, and they had two sessions of the immediate response task and two sessions of the delayed response task on a PC each time. The parameters of the training tasks were the same as those of the onboard training, and each session took 5 min. For the delayed response task, the required delay for the left/right-button response to the periodic stimuli was set to *n* = 1 for the first 12 training days and to *n* = 2 for the later 12 training days. At the end of each training session, the system gave visual feedback that informed the subjects of the transition of performance.

#### 2.5.3. Group C: Crossword Training Group

*Crossword Training Task*. We adopted a number crossword puzzle as a training task for Group C. This is a version of a kanji crossword puzzle with numbers in which squares with the same number should contain the same kanji character. Some cells are filled with kanji characters in the initial state, and a set of available characters is also provided. There are no additional clues. The goal is to fill all the white numbered squares so that every succession of white squares makes a meaningful word. We used a PC program that was played using a computer mouse.

For successful performance, the task was supposed to require the ability to make inferences, processing speed, and executive function in order to efficiently try various possibilities, working memory to keep in mind the possible choices and hypotheses, and divided attention to check the consistency of the consequences at different positions simultaneously. Vocabulary was also required; however, it was not critical as the answers consisted of words that most people know.

*Training Procedure*. Each of the subjects in Group C visited a room in our institute, and they were trained on solving the number crossword puzzle on a PC program for 20 min each time. All the operations were performed using a computer mouse. Their progress was recorded, and if they could not finish a problem in the 20 min, they continued it the next time they came in. If they completed a problem before the time limit, they proceeded to the next problem. If they gave up on a problem, the answer was shown, and they then proceeded to the next problem.

### 2.6. Neuropsychological Tests

To evaluate the possible effects of the three types of cognitive training on a broad range of cognitive functions, the subjects underwent the following neuropsychological tests in the Pre and Post tests: MMSE [40], the Block Design (BD) subtest in the Wechsler Adult Intelligence Scale (WAIS) III [43], the Frontal Assessment Battery (FAB) at bedside [44], the Word Fluency Test (WFT) [45], the Trail Making Test (TMT) [46], the Symbol-Digit Modalities Test (SDMT) [47], the Spatial Span subtest of the Wechsler Memory Scale-Revised (WMS-R) [48], the Benton Visual Retention Test (BVRT) [49], the Rey-Osterrieth Complex Figure Test (CFT) [50], the Rey Auditory-Verbal Learning Test (AVLT) [51], and the Judgment of Line Orientation (JLO) [52]. The MMSE was used to screen for cognitive impairment, and subjects were required to have a score of 25 or more for inclusion in the analyses (no subjects were excluded). The other test measures were used to evaluate cognitive functions, as explained in the next section.

### 2.7. Analyses of Cognitive Functions

One way to obtain a general and stable measure of a cognitive construct is to calculate a composite measure of multiple neuropsychological tests relevant to the cognitive function [5, 37, 53, 54]. By summing up the standardized *t* scores (mean, 50; standard deviation, 10) of the relevant test measures explained above with some required sign-flipping so that all the scores indicated better performance when they were higher, we evaluated the following three composite measures for the specific cognitive function domains: (1) processing speed composite, which included TMT-A and SDMT; (2) executive function composite, which included FAB, WFT, and TMT-B; and (3) working memory composite, which included SS, BVRT, and AVLT. As stated above, these cognitive domains were among the main targets of the onboard cognitive training. Therefore, we hypothesized that Group V as well as Group P would show significant improvements in these cognitive domains. In addition, by combining BD, WFT, TMT-B, BVRT, CFT-Copy, CFT-Recall, AVLT, and JLO, we calculated a composite measure of cognitive impairment (COGSTAT), which has been reported to be a significant predictor of driving safety in drivers with Alzheimer's disease [5, 53, 55].

The improvements (Post − Pre) in each composite measure were computed for each subject. To exclude the possible influence of preexisting factors of noninterest, the improvement values of all subjects were fit to a general linear model with their mean-centered age, sex, and Pre scores (baseline) as explaining variables, and they were adjusted for these confounding variables. Then, the significance of the training effects on the improvements in each composite within each intervention group was tested with the Wilcoxon signed-rank test (one-sided with the alternative hypothesis being that the improvement is positive). We used the nonparametric Wilcoxon test because some of the outcome variables did not satisfy the assumption of normal distribution on which the parametric *t*-test is based. Because of the exploratory nature of this study, the test results were considered significant at *P* < 0.05 with the Benjamini-Hochberg (BH) procedure [56] applied to the combined data of the cognitive composites and the driving safety for each group in order to control the false discovery rate (FDR).

In addition, for each pair of groups, we conducted the Wilcoxon rank-sum test (two-sided) in order to explore the significant group differences in the improvements in the composites. The test results were considered significant at *P* < 0.05 with the BH in order to control the FDR.

### 2.8. MRI Image Acquisition

All MRI images were collected using a 3-T Philips Intera Achieva scanner. Using a magnetization-prepared rapid gradient-echo (MPRAGE) sequence, high-resolution T1-weighted structural images [240 × 240 matrix; repetition time (TR): 6.5 ms; echo time (TE): 3 ms; inversion time (TI): 711 ms; field of view (FOV): 24 cm; 162 slices; slice thickness, 1.0 mm] were collected. The scan time was 8 min 3 s. Diffusion-weighted data were acquired with a spin-echo echo planar image (EPI) sequence [TR: 10,293 ms; TE: 55 ms; big delta (Δ): 26.3 ms; little delta (*δ*): 12.2 ms; FOV: 22.4 cm; 2 × 2 × 2 mm^3^ voxels; slice thickness, 2 mm; 60 slices; sensitivity-encoding (SENSE) reduction factor, 2; number of acquisitions, 1]. The diffusion weighting was isotropically distributed along 32 directions (*b* value, 1,000 s/mm^2^). In addition, a data set with no diffusion weighting (*b* value, 0 s/mm^2^) was acquired. The total scan time was 7 min 17 s.

### 2.9. Analysis of Brain Structural Changes

#### 2.9.1. Voxel-Based Morphometry Analysis of Regional Gray Matter Changes

To examine the effects of the different types of training interventions on the rGMV, we performed VBM analyses on the T1-weighted structural images.

First, the T1-weighted images were processed with SPM8 software (Wellcome Department of Cognitive Neurology, London, UK; http://www.fil.ion.ucl.ac.uk/spm/software/spm8/) with the Diffeomorphic Anatomical Registration using Exponentiated Lie Algebra (DARTEL) [57] method in MATLAB (The MathWorks, Inc., Natick, MA, USA). The New Segmentation algorithm was applied to all T1-weighted images both before and after the interventions to extract tissue maps that corresponded to gray matter (GM), white matter (WM), and cerebrospinal fluid (CSF). To reduce the risk of missegmenting irrelevant tissues, such as dura, into GM, we used a modified gray matter tissue probability map (TPM) that was obtained by changing the values of the voxels of the standard GM TPM in SPM8 with values less than 0.25 to zero. The GM tissue images obtained were then subjected to the DARTEL template creation tool. A study sample-specific template and the nonlinear deformations that best aligned the images with the template were obtained by iteratively registering the imported images with their averages. The DARTEL normalization tool was used to apply the estimated deformations and spatially normalize the tissue images to the template and then to the Montreal Neurological Institute (MNI) standard space with a 12-parameter affine linear transformation. The warped images were modulated by the Jacobian determinants that were derived from the nonlinear deformation parameters to compensate for individual local volume deformations [58]. Then, all the warped and modulated GM images were smoothed by convolving a 12 mm full-width at half maximum isotropic Gaussian kernel. Finally, the signal changes in the rGMV between the Pre and Post images were computed at each voxel for each subject. In this computation, we included only the voxels that showed rGMV values >0.10 in both the Pre and Post images in order to avoid possible partial volume effects around the tissue borders.

The processed maps representing the rGMV changes between the Pre and Post scans (rGMV Post − rGMV Pre) were then forwarded to the whole-brain group level analysis. Within each intervention group, we tested the rGMV changes (both positive and negative) with a general linear model (one sample *t*-test with covariates). In addition, for each pair of groups, we performed one-way analysis of covariance (two-sample *t*-tests with covariates) in order to find the significant differences in the ways that the rGMV changed between the groups. In both types of tests, the mean-centered age, sex, total gray matter volume in the Pre scan, and total intracranial volume in the Pre scan were included in the model as covariates. The statistical model estimation was performed with SPM8, and the level of statistical significance was set at *P* < 0.05 with family-wise error corrected for multiple comparisons with the nonisotropic adjusted cluster level [59] with an underlying voxel level of *P* < 0.001. For the correction, the NS toolbox (http://fmri.wfubmc.edu/cms/software#NS) was used. Nonisotropic adjusted cluster-size tests can and should be applied when cluster-size tests are used for data that is known to be nonstationary (in other words, not uniformly smooth), just as is the case for VBM data [59].

#### 2.9.2. Tract-Based Spatial Statistics on Diffusion Tensor Images

The preprocessing and tract-based spatial statistics (TBSS) analyses of the DTI data were performed with the FSL software package (http://www.fmrib.ox.ac.uk/fsl/, Version 5.0), according to the standard procedures described previously in [39]. First, the DTI images of each subject and each time point (Pre and Post) were prealigned with each other in order to correct for eddy current, distortion, or head motion. Then, the diffusion tensor was calculated and used to obtain an FA map, and nonbrain voxels were excluded. The obtained FA maps were subjected to noise reduction by eroding exterior noise-susceptible voxels, aligned to the FMRIB58_FA MNI standard-space image (1 × 1 × 1 mm^3^) by nonlinear registration and averaged to obtain a study-specific mean FA image. A tract skeleton was generated from the mean FA image, and it was thresholded for FA values more than 0.2 in order to restrict further analysis to points within the white matter that had been successfully aligned across subjects. All individual FA maps were projected to the thresholded mean FA skeleton, resulting in the skeletonized FA data for each subject and time point. The projection to the mean FA skeleton is the essence of the TBSS analysis to reduce potential problems of misregistration as a source of false-positive or false-negative results in the subsequent voxel-wise cross-subject statistics [39].

In the same manner as the VBM analysis on the rGMV, the changes in the projected FA signals between Pre and Post (FA Post − FA Pre) were computed for each subject at each voxel in the mean FA skeleton and subjected to statistical tests of the effects of the interventions. We conducted a one-sample test of the FA changes within each group and a two-sample test of the differences in the FA changes between each pair of two groups. Mean-centered age and sex were included in the model as covariates. The statistical model estimates were performed with permutation-based cross-subject statistics [60] implemented as the Randomise Tool in FSL. A total of 5,000 permutations were performed for each contrast, and statistical inferences were made with the threshold-free cluster enhancement (TFCE) [61] correction for multiple comparisons. Fully corrected *P* values less than 0.05 were considered significant.

### 2.10. Driving Safety Tests

#### 2.10.1. Driving Aptitude Tests with a Simulator Test Unit

Subjects were tested with the CG400 driving aptitude test unit (TKK 7020, Takei Scientific Instruments Co., Ltd., Niigata City, Japan) [62]. This computer-based driving ability test unit has been licensed by the National Police Agency in Japan, and it has been installed in many driving schools. It is widely used as an evaluation system for the driving aptitude of elderly people. The test unit is equipped with a cathode-ray tube monitor, a steering wheel, an accelerator, and brake pedals, and it is in accordance with the National Police Agency System Guidelines [63]. It provides tests on the following four types of tasks: simple response, selective response, handling operation, and divided attention to multiple tasks. Based on the performances of these tasks, the test unit evaluates five-level grades on 14 driving-related aspects: (1) speed of simple responses, (2) stability of simple responses, (3) accuracy of selective responses, (4) speed of selective responses, (5) stability of selective responses, (6) accuracy of steering operations, (7) balance of steering operations, (8) efficiency of steering operations, (9) divided attention accuracy to the center of the visual field, (10) divided attention speed to the center of the visual field, (11) divided attention stability to the center of the visual field, (12) divided attention accuracy to the peripheral visual field, (13) divided attention speed to the peripheral visual field, and (14) divided attention stability to the peripheral visual field. The total grade obtained by summing up the 14 grades (max = 70) gives an integrated evaluation of driving aptitude. The test took an average of 25 min, including the explanations.

#### 2.10.2. On-Road Evaluation by Driving School Instructors

Subjects visited a local driving school and had a driving test before and after the intervention period. The tests were scheduled on a day different from the days when the other parts of the Pre/Post tests were conducted at our institute but within a week before the start of each subject's intervention period (Pre) and within a week after the end of the intervention period (Post). The tests were conducted during the daytime but not on days with extremely bad weather with limited view or with snow cover on the roads.

The driving test was conducted in the training course of the driving school. Subjects drove a 1.7 km route with various safety check points three times, taking 20 min in total. One of the two driving instructors who acted as tester was seated on the passenger seat during the test and silently observed the driving behavior. The instructor counted the risky actions made by the subject. The risky actions were identified according to the 26 check items and classifications listed in Table 3, and the counts were converted to a five-level grade for each check item, resulting in a total grade of a maximum of 130 for driving with no unsafe actions counted.

### 2.11. Analysis of Driving Safety Changes

The total grade given by the driving aptitude test unit was analyzed in the same manner as the cognitive composite measures: improvements (Post − Pre) of the total grade were computed for each subject and adjusted for age, sex, and Pre (baseline) total grades. The within-group training effects on the improvements were tested with the Wilcoxon signed-rank test (one-sided with the alternative hypothesis being that the improvement is positive). The between-group differences in the improvements were tested with the Wilcoxon rank-sum test (two-sided).

The total grade of the on-road driving safety test was first adjusted for the effect of the testers (driving instructors). Then, the data were subjected to the same within-group and between-group analyses as the driving aptitude measure.

## 3. Results

### 3.1. Performance Progress in the Training Tasks

As explained in the Materials and Methods, the training tasks of Groups V and P consisted of adaptive training in which the tempo of the presentation of the stimuli (trials) was dynamically adjusted according to the subject's performance level. Hence, the numbers of presented and correctly processed stimuli worked as indicators of the performance progress of the trained tasks. Subjects in both Groups V and P showed sustained progress in the performances of the two training tasks.

In Group C, we did not have an exact quantitative evaluation of the improvements in the ability to perform the training task because the crossword puzzle problem solved by each subject differed day by day according to his/her progress. However, the recordings of the time keepers confirmed that all the subjects made progress so that their answer characters were found more quickly and steadily.

### 3.2. Changes in Cognitive Function

To characterize the effects of each training intervention on cognitive function, we conducted within-group tests of the improvements in the composite measures of three cognitive domains (processing speed, executive function, and working memory) as well as in a composite measure of cognitive impairment (COGSTAT) [5]. The changes in the composite scores (Post − Pre) were adjusted for age, sex, and the Pre (baseline) score, and the hypothesized improvements (Post − Pre > 0) were tested using the one-sided Wilcoxon signed-rank test for each group, with the BH procedure [56] employed to correct for multiple comparisons.

**Figure 2:**
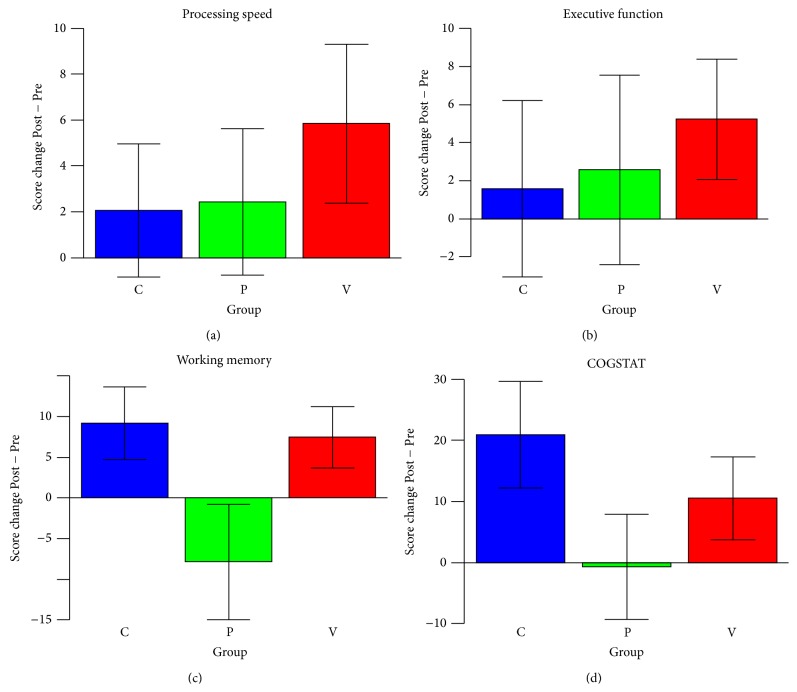
Changes in the cognitive function composite scores in each training group. (a) Processing speed was evaluated as a composite of the Trail Making Test- (TMT-) A and Symbol-Digit Modalities Test (SDMT) measures. (b) Executive function was evaluated as a composite of the Frontal Assessment Battery (FAB), Word Fluency Test (WFT), and TMT-B test measures. (c) Working memory was evaluated as a composite of the Spatial Span (SS), Benton Visual Retention Test (BVRT), and Auditory-Verbal Learning Test (AVLT) measures. (d) Composite measure of cognitive impairment (COGSTAT) [5] was evaluated by combining the Block Design (BD), WFT, TMT-B, BVRT, Complex Figure Test- (CFT-) Copy, CFT-Recall, AVLT, and Judgment of Line Orientation (JLO) scores. Score changes were adjusted for age, sex, and Pre (baseline) scores. The error bars in the graphs show the standard errors of the mean (SEM) for the subjects in each group.

Figure 2 and Table 4 show the summaries of the intervention effects and their significance. Group V showed significant improvements in the processing speed (*P* = 0.048) and working memory (*P* = 0.048) composites as well as marginally significant improvements in the executive function (*P* = 0.076) and COGSTAT (*P* = 0.076) composites. Group P showed no significant improvements in any of the composites. Group C showed no significant improvements in any of the composites, but marginally significant improvements in the working memory (*P* = 0.092) and COGSTAT (*P* = 0.078) composites.

In addition, we explored the intergroup differences in the improvements in each cognitive domain with the two-sided Wilcoxon rank-sum tests between each pair of groups, with the BH procedure used for multiple comparison corrections. The statistical tests found no significant intergroup differences for any of the cognitive composites (Table 4; Figure 2).

### 3.3. Changes in Brain Structure

#### 3.3.1. Regional Gray Matter Volume Changes

**Figure 3:**
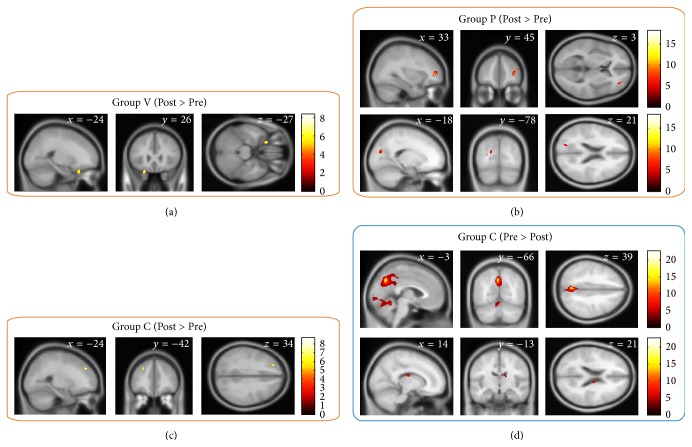
Group V showed significant rGMV increases in the left inferior frontal gyrus (a). Group P showed significant rGMV increases in the right middle frontal gyrus and the left superior occipital gyrus (b). Group C showed significant rGMV increases in the left dorsolateral prefrontal cortex (c) and significant rGMV decreases in the precuneus, the medial cerebellum, and the right caudate (d). The colored clusters show regions with rGMV changes that were significant with a family-wise error corrected *P* < 0.05 at the nonisotropic adjusted cluster level [59], with an underlying voxel level of *P* < 0.001.

With the VBM analysis of the one-sample *t*-tests of the (Post − Pre) differences in the images in each training group, we found several significant rGMV changes in each group. In Group V, significant rGMV increases were observed in the left orbitofrontal cortex (OFC) adjacent to the inferior frontal gyrus (IFG; *t* = 8.33, *P* = 0.033; Figure 3(a)). In Group P, significant rGMV increases were observed in the right middle frontal gyrus (MFG; *t* = 18.24, *P* = 0.003) and the left superior occipital gyrus (SOG; *t* = 7.25, *P* = 0.025; Figure 3(b)). In Group C, significant rGMV increases were observed in the left dorsolateral prefrontal cortex (DLPFC; *t* = 8.70, *P* = 0.033; Figure 3(c)) and significant rGMV decreases were observed in the precuneus (*t* = 22.65, *P* = 0.002), the medial cerebellum (*t* = 12.10, *P* = 0.002), and the right caudate (*t* = 8.76, *P* = 0.039; Figure 3(d)). No significant changes were observed for the other within-group contrasts.

**Figure 4:**
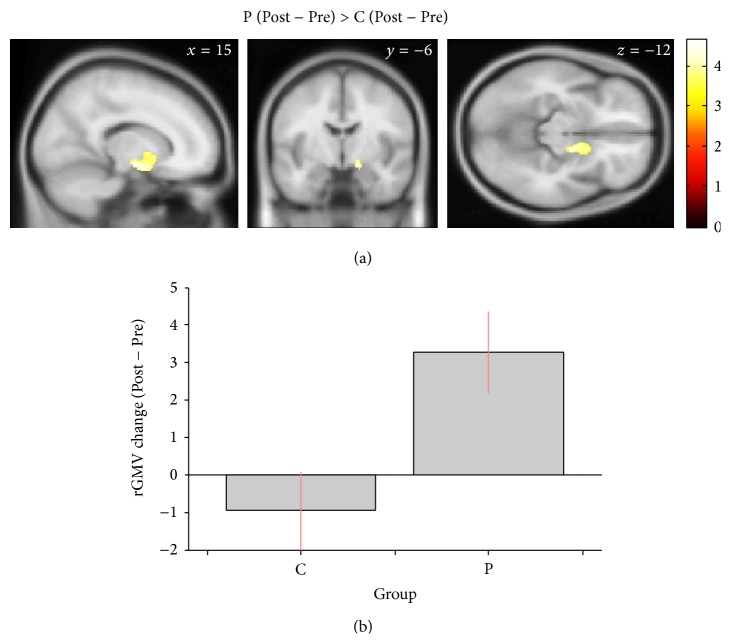
Significant between-group difference in regional gray matter volume (rGMV) changes. (a) In the structures around the right caudate, the rGMV changes were significantly different between Groups P and C. The colored cluster shows the region that exhibited significant differences in rGMV change with family-wise error corrected *P* < 0.05 at the nonisotropic adjusted cluster level [59] with an underlying voxel level of *P* < 0.001. (b) The average of rGMV changes in each group shows that the differences in the region were largely because of the rGMV increases in Group P. The error bars in the graph show the 95% confidence intervals.

In addition, two-sample *t*-tests that compared the rGMVs between the pairs of groups revealed that the rGMV increases in the structures around the right caudate were significantly larger in Group P than in Group C (Figure 4). No significant differences in rGMV changes were observed for the other between-group contrasts.

The summary of these rGMV changes is given in Table 5.

#### 3.3.2. Fractional Anisotropy Changes in White Matter

**Figure 5:**
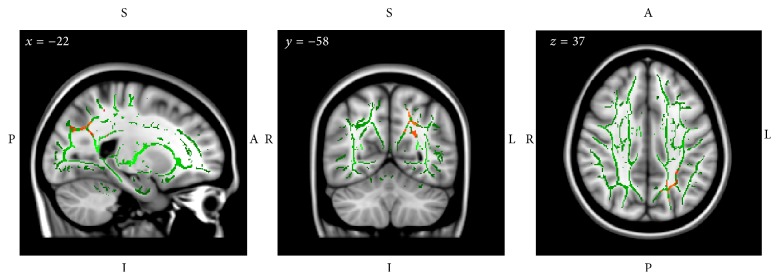
Tract-based spatial statistical analysis in Group C detected significant increases (Post and Pre) in fractional anisotropy (FA) in the left posterior white matter. Gray: MNI152 T1 template image, green: intergroup average white matter skeleton, and red-yellow: voxels that show threshold-free cluster enhancement-corrected significant (*P* < 0.05) increases in FA in Group C. Note that the images are displayed according to radiological convention, with the right hemisphere on the left.

TBSS analysis was used to investigate the effects of the different types of training on white matter integrity, and we conducted one-sample tests of the FA changes (FA Post − FA Pre) within each group. The significance of the permutation test was corrected for multiple comparisons with the TFCE correction [61], and clusters determined by the threshold (1 − *P*) > 0.95 (or equivalently *P* < 0.05) on the corrected *P*-maps were considered significant. The results of the TBSS analysis revealed significant FA increases in white matter tracts around the left intraparietal sulcus (IPS) and the left precuneus of Group C (Figure 5; Table 6). No significant increases or decreases of FA were observed in the other training groups.

In addition, we conducted two-sample tests of the differences in the FA changes between each pair of groups, and we found no significant differences in the FA changes.

### 3.4. Changes in Driving Safety Measures

The total grades obtained from the driving aptitude test unit were analyzed in the same way as the cognitive composite measures: the (Post − Pre) total score changes were adjusted for age, sex, and the Pre score, and the hypothesized improvements (Post − Pre > 0) were tested with one-sided Wilcoxon signed-rank tests for each group, with the BH correction used for multiple comparisons. The total grades for the on-road driving safety tests were analyzed with the same procedure after they had first been adjusted for the effects of the testers. Moreover, the intergroup differences in the improvements in driving aptitude and the on-road driving safety evaluations were also tested in the same manner as the cognitive composites with the two-sided Wilcoxon rank-sum test between each pair of groups, with the BH procedure used for multiple comparison corrections.

**Figure 6:**
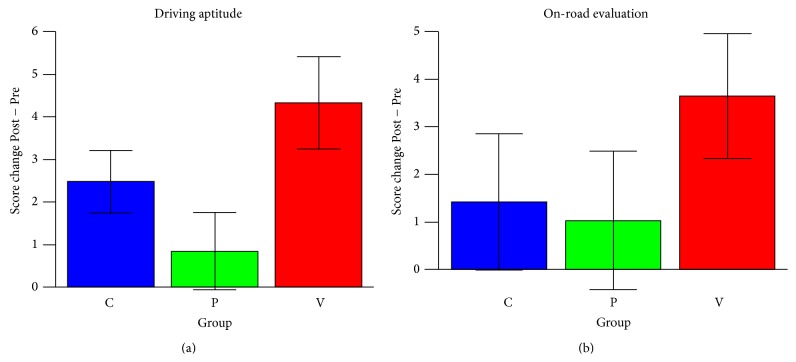
Changes in driving safety measures in each training group. The measures are as follows: (a) the total grades obtained from the driving aptitude test unit and (b) the total grades obtained from the on-road driving safety test. The score changes were adjusted for age, sex, and Pre (baseline) scores. The error bars in the graphs show the standard errors of the mean (SEM) of the subjects in each group.

Figure 6 and Table 4 show the summaries of the intervention effects on the driving safety measures. Group V showed significant improvements in the driving aptitude (*P* = 0.015) and on-road evaluation (*P* = 0.040). Group P showed no significant improvements in either test. Group C showed significant improvements in driving aptitude (*P* = 0.015) but not in the on-road evaluation. The between-group differences in the driving safety measures were not significant.

## 4. Discussion

The purpose of the present intervention study was twofold. The first aim was to verify the effectiveness of the newly developed onboard cognitive training system in enhancing the driving safety of elderly people as well as its effects on related basic cognitive functions and neural plasticity. The other aim was to elucidate the diverse effects that the different types of cognitive training exerted on cognitive functions, brain structure, and driving safety by observing changes in these aspects simultaneously. The results were generally in accordance with these aims and the expectations. Group V, who underwent onboard training, showed significant improvements in the driving safety measures as well as significant or marginally significant improvements in multiple cognitive domains, supporting the expected beneficial effect of the training. The MRI data analyses of neural plasticity detected structural changes that seemed to be associated with the different functional demands for each training task. In the following sections, we discuss each aspect of the training-induced changes in detail.

### 4.1. Changes in Cognitive Functions

Group V with the onboard training showed significant improvements in the processing speed and working memory composites as well as marginally significant improvements in executive function. These results were consistent with our expectations because the training program focused exactly on these cognitive domains which are relevant to driving safety [4], along with visual processing and attention [6, 22].

The results of Group P, who showed no significant improvements in either of the cognitive domains, were less expected. From the shared basic design of the on-PC training of Group P and the onboard training of Group V, we assumed that the involvement of processing speed, executive control, and working memory functions would be at the same level. In addition, the performance changes in the trained tasks were also similar between the two groups. Therefore, we did not anticipate the qualitative differences in the training effects of Groups V and P on these basic cognitive function domains. One interpretation of these results is that cognitive training can have greater effects if it also involves bodily performance, which the onboard training contained but the on-PC training lacked. This interpretation was consistent with the results of studies that have shown positive effects of aerobic exercise [16, 18, 19, 64] or even very mild physical activity [65] and effects of the combination of cognition and aerobic training [20] on cognitive functions and neural plasticity. Another possible explanation is that the differences in the ease of operation (or the familiarity to the user interface) and the resultant differences in the comfort, motivation, and engagement in the task caused differences in the transfer effects of the training to cognitive functions. For example, the speed of processing training, which has been reported to have an enhancing effect on cognitive and daily functions [8–11], uses a PC with a touch screen instead of a computer mouse and thus makes the training more intuitive and easier, particularly for those who are not familiar with the computer mouse operation. Yet another possible explanation is that our subjects had higher baseline functioning and thus relatively lower sensitivity to training. Studies have observed stronger training effects for participants with relatively poor performance at baseline, suggesting that those with reduced functioning were likely to receive greater benefit from training [8, 11]. In either case, the results indicated an advantageous effect of implementing cognitive training onboard, at least for elderly drivers.

Group C trained to solve crossword puzzles showed marginally significant improvements in the working memory as well as the COGSTAT composites. These results seemed to support the findings of previous reports that daily intellectual activity, such as puzzle solving, has beneficial effects on cognitive function in the healthy elderly [23–26]. We also noted the possibility that the training setting for Group C in this study, which required concentrated effort to solve the puzzles within the limited time frame of 20 min, may have entailed larger effects than solving such puzzles as a daily habitual activity [27]. The observation that the crossword puzzle training had marginal effects on working memory as well as on the COGSTAT, which combined memory-related test measures such as CFT-Recall, AVLT, and BVRT, was understandable because of the nature of the task. The number crossword puzzle used in the training required the memorization and retrieval of various types of information, such as the available characters to fill in the squares, the characters already filled in that work as constraints to make meaningful words, and the possible choices and hypotheses in parallel.

### 4.2. Structural Changes in the Brain

Group V showed significant rGMV increases in the left OFC adjacent to the IFG. This region has been frequently implicated in sensory integration, the processing of affectively salient stimuli, reward prediction, and decision making [66]. These findings may support the speculation that the onboard training task successfully elicited motivated engagement, leading to better transfer effects for a wide range of cognitive domains. In contrast, it has been reported that the rGMVs of the bilateral OFC are negatively correlated with individual differences in impulsivity, suggesting the region's contribution to executive function [67]. In addition, gray matter volumes in the bilateral IFG have been reported to positively correlate with individual differences in processing speed in healthy elderly [68]. The observed rGMV increases may have reflected improvements in these functions.

The regions that showed significant rGMV increases in Group P may correspond to the functional processes required by the training task. For example, the left SOG was involved in the visual processing of stimuli, and the right MFG integrated the bottom-up processing with the top-down control [69]. Although the direct intergroup comparison did not show significant differences, the different patterns of the rGMV changes between Groups V and P suggested that the two groups may have engaged in the training in different ways, despite similarity in the basic task design.

Group C showed the widest range of neural plasticity that was largely reasonable considering the nature of the training task and its observed effects on working memory. The left DLPFC, which showed significant rGMV increases in Group C, constitutes the frontoparietal network essential for working memory [70–72]. The significant FA increase in the white matter tracts around the left IPS and the left precuneus that was revealed by the TBSS analysis was consistent with previous findings that working memory training induces significant FA increases in younger adults [38]. In addition, it has been reported that the FA values in the same WM region positively correlate with processing speed [73]. Interestingly, the regions that showed significant rGMV decreases, including the precuneus, the medial cerebellum, and the right caudate, have also been reported to be involved in working memory [74–76]. Recent findings, although largely in younger cohorts, have suggested that, in certain cases, rGMV decreases rather than increases are associated with increased cognitive and behavioral functions [31, 33, 77]. Therefore, it is possible that these rGMV decreases also reflected the training-induced cognitive enhancements. Another possible explanation for the observed rGMV decreases may be attributed to age-induced shrinkage. In this context, it has been reported that the cerebellum and caudate are susceptible to age effects [78] but that the precuneus is less affected by age [79]. Considering the intervention period of 2 months, it is not likely that all the observed rGMV decreases in Group C were caused by an aging effect. Further studies are needed to clarify this point.

### 4.3. Changes in Driving Safety

Group V showed significant improvements in both of the driving safety measures, the driving aptitude test, and the on-road evaluation. These results indicated that the onboard cognitive training system indeed had a transfer effect on the ability to drive safely. In contrast, Group P did not show significant improvements in either of the measures. These results were consistent with our initial hypothesis that the differences in the driving-related factors of the training tasks, such as the required field of view for the processing of stimuli and the motor control involving distributed body parts, would make the onboard training more effective than the on-PC training. Another explanation is that the training effects on the basic cognitive domains of processing speed, executive function, and working memory, which were significant only in Group V, were transferred to driving safety. This interpretation may again explain the discrepancy from the studies which showed that driving safety improvements could be driven with computer only methods [8–11]: our subjects in Group P who lacked significant training effects on the basic cognitive functions, possibly due to the difficulty of operation as discussed above, may also have lacked the transfer effects to driving safety. Note that all the subjects were daily drivers who reportedly owned a car and who drove more than three times a week on an average. Therefore, it is not likely that simple contact with the driving environment in Group V induced the difference. Either way, the results again suggested the advantage of an onboard training system over a general (on-PC) training of a similar type.

Group C showed significant improvements in the driving aptitude measures but not in the on-road driving safety evaluation. This can also be interpreted in terms of the difficulty of the far-transfer effect. From its purpose as an aptitude evaluation system, the driving aptitude test continuously poses situations with high cognitive demands, and thus, the improvements in the general cognitive function domains would have better benefitted the evaluation. In comparison, the on-road evaluations involved more factors specific to the domain of driving. It has been repeatedly reported that the far-transfer effects from training to a task with less similarity are harder to achieve than the near-transfer effects from training to a task with larger overlap in the underlying processes, especially for older adults [80–85]. Therefore, the effects of crossword training on working memory and other cognitive functions may have been better transferred to the “nearer” driving aptitude tests and not to the “farther” on-road evaluations.

### 4.4. Limitations

The major limitation of this study was the small sample size, which was mainly because of the relatively limited resources for the requirements of isolation in order to prevent interactions between the subjects, supervision, and control in the training intervention. Consequently, we had only limited results from the intergroup analyses, which would have been more conclusive if they had been significant, compared with the within-group results reported in the present study. Therefore, a possible future direction would be to replicate and extend the results of the present study with a larger sample and less strict (more casually controlled) trial design. One might argue that another way to establish the intergroup effects is by introducing a no-intervention control group. However, this would be less helpful because comparisons with a no-intervention control cannot dissociate the confounding effects from participation in the trial itself. In addition, we explored the direct association between the cognitive enhancements, neural plasticity, and the changes in driving safety with correlational analyses, as has been reported in a cross-sectional study [86]; however, we did not find any significant relationships (not shown in this paper). To determine if this was due to insufficient statistical power or if this originated from the complexity of the relationships of cognitive function through the substrate to the driving behavior [7], a study with a larger sample is required.

Another remaining issue is the maintenance of the training effects. How long are the effects of the training sustained once the training is terminated? How effective is the addition of booster training and what is the best timing [87, 88]? Additional follow-up studies are required to answer these questions.

### 4.5. Real-World Applicability of the Onboard Cognitive Training

As described in the Materials and Methods, the onboard cognitive training system was developed with the expectations that it can be utilized by the elderly daily drivers easily and continuously in a natural extension of daily driving activities and can facilitate the transfer of training effects to actual driving situations. The obtained results generally supported the usefulness of the onboard training.

Regarding the installation of the onboard system, it can be largely covered by existing or commonly available equipment. The training programs can be implemented on a smartphone or on a computer of an automotive navigation system. The information of the steering wheel and brake pedal can be collected through a standardized vehicle network bus, which has been equipped in most of modern cars. Audio speakers are also equipped in most of cars. Although the LED lights and their controller need to be newly introduced, they are relatively inexpensive. In total, we expect that the onboard training program can provide a viable option for enhancing elderly drivers' safety in real life.

## 5. Conclusions

To the best of our knowledge, this is the first study to simultaneously investigate the possible effects of different types of cognitive training on cognitive enhancement, neural plasticity, and changes in driving safety. The results showing improvements in cognitive functions and driving safety verified the effectiveness of the onboard training system that we developed and suggested that implementing a training system in the car facilitates enhancements of driving safety. However, the results of the behavioral improvements induced by crossword puzzle training, which had less apparent relevance to driving, also implied potential usefulness. Combined with the neural plasticity results from the structural analyses of the MRI data, these different types of training can contribute to driving safety through different underlying mechanisms. Thus, one future direction worth exploring is a multimodal training approach [89] that combines these different types of training. Despite the limitations discussed above, we believe that the reported results and implications provide useful insights for future studies on the enhancements of driving safety and broad daily activities of elderly people.

## Supplementary Material

Supplementary Materials provide additional information and data on the cognitive training tasks and tests.

## Figures and Tables

**Table 1:** Baseline characteristics of the subjects.

	Group C		Group P		Group V		Main effect of group	
	*n* = 12 (4 F/8 M)		*n* = 11 (4 F/7 M)		*n* = 12 (4 F/8 M)			
	Mean	SD	Mean	SD	Mean	SD	*F*-value	*P* value
Age (year)	67.77	4.67	68.24	5.66	67.67	4.57	0.042	0.959
Education (year)	14.50	1.73	13.18	2.23	13.42	2.43	1.257	0.298
MMSE base (score)	28.83	0.94	28.27	1.74	28.33	1.50	0.552	0.581

The base score indicates the preintervention test score. The main effect of group was tested with one-way analysis of variance (ANOVA). Group C trained with crossword puzzles. Group P trained with an on-PC cognitive training program. Group V trained in a vehicle with an onboard cognitive training program. F: female; M: male; SD: standard deviation; Education: number of years of education completed; MMSE: Mini-Mental State Examination.

**Table 2:** Relationship between the difficulty levels and the periods of rhythmic stimuli in the immediate response task and delayed response task.

Levels	1	2	3	4	5	6	7	8	9	10

Period (s)	3.0	2.0	1.5	1.2	1.0	0.9	0.8	0.7	0.6	0.5

**Table 3:** Unsafe driving actions checked in the on-road evaluation.

Scene	Checked item	Unsafe actions	Count to evaluation mapping
Right turn	Turn signal Steering Safety check Safe speed	Fail/too late Too tight/too loose/unstable Fail/insufficient Too fast	0 → 5, 1 → 4, 2-3 → 3, 4-5 → 2, ≥6 → 1

Left turn	Turn signal Steering Safety check Safe speed	Fail/too late Too loose/unstable/swinging opposite Fail/insufficient/forgot to look back Too fast	0 → 5, 1-2 → 4, 3-4 → 3, 5-6 → 2, ≥7 → 1

Passing blind intersection	Fail to stop Safety check	No slowing/going slow Fail/insufficient	0 → 5, 1 → 4, 2 → 3, 3 → 2, ≥4 → 1

Intersection requiring stop	Fail to stop Stop position Safety check	No slowing/going slow Intrusion into intersection/crossing stop line Fail/insufficient	0 → 5, 1 → 4, 2 → 3, 3 → 2, ≥4 → 1

Changing lanes	Signaling Safety check Steering	Fail/too late Fail/insufficient Abrupt	0 → 5, 1 → 4, 2-3 → 3, 4-5 → 2, ≥6 → 1

Passing beside a stopped car	Signaling Safety check Safe speed Distance Steering	Fail/too late Fail to check front/fail to check side/inattention Too fast Too close Abrupt	0 → 5, 1 → 4, 2 → 3, 3 → 2, ≥4 → 1

Curve	Course Safe speed Safety check	Too inside/too outside Too fast Fail/insufficient	0 → 5, 1-2 → 4, 3-4 → 3, 5-6 → 2, ≥7 → 1

Other	Priority at intersection	Miss	0 → 5, 1 → 4, 2 → 3, 3 → 2, ≥4 → 1

**Table 4:** Summary of the changes in the cognitive function composites and the driving safety measures within each training group and the statistical differences between the groups.

	Group C (*n* = 12)			Group P (*n* = 11)			Group V (*n* = 12)			P versus C	V versus C	V versus P
	Mean	SEM	*P* value	Mean	SEM	*P* value	Mean	SEM	*P* value	*P* value	*P* value	*P* value
Cognitive function composites												
Processing speed	2.05	2.89	0.455	2.44	3.19	0.520	5.84	3.46	0.048	0.833	0.341	0.391
Executive function	1.58	4.63	0.455	2.58	4.98	0.520	5.23	3.16	0.076	0.786	0.786	0.786
Working memory	9.20	4.45	0.092	−7.86	7.09	0.926	7.47	3.78	0.048	0.156	0.713	0.156
COGSTAT	20.89	8.73	0.078	−0.67	8.62	0.621	10.53	6.76	0.076	0.354	0.413	0.413
Driving safety measures												
Driving aptitude	2.48	0.74	0.015	0.85	0.90	0.520	4.33	1.08	0.015	0.219	0.219	0.112
On-road evaluation	1.42	1.43	0.255	1.03	1.45	0.525	3.64	1.31	0.040	0.695	0.567	0.567

SEM: standard error of the mean; COGSTAT: a composite measure of cognitive impairment.

The means and SEMs of each of the changes in the measures were calculated from the differences in the measure (Post − Pre) that were adjusted for age, sex, and the Pre (baseline) score of the measure. The *P* values for each group show the significance of the improvements (Post − Pre > 0) obtained from the Wilcoxon signed-rank tests (one-sided) and corrected by the Benjamini–Hochberg (BH) procedure to control the false discovery rate (FDR). The *P* values for each pair of the groups were from the Wilcoxon rank-sum test (two-sided), which explored the significant group differences in the improvements of each measure with a correction for multiple comparisons with the BH procedure in order to control the FDR.

**Table 5:** Summary of regional gray matter volume changes detected by the voxel-based morphometry analysis.

Area	MNI peak coordinates (mm)				*t* value	Cluster	
		*x*	*y*	*z*		Raw size (mm^3^)	*P* value

Increase (Post > Pre) in Group V							
OFC/IFG	L	−24	26	−27	8.33	311	0.033
Increase (Post > Pre) in Group P							
MFG	R	33	45	3	18.24	807	0.003
SOG	L	−18	−78	21	7.25	358	0.025
Increase (Post > Pre) in Group C							
DLPFC	L	−24	42	34	8.70	192	0.033
Decrease (Pre > Post) in Group C							
Precuneus	L	−3	−66	39	22.65	6446	0.002
Cerebellum	L	−6	−78	−24	12.10	4516	0.002
Caudate	R	14	−13	21	8.76	196	0.039
Larger increase in Group P compared with Group C							
Extranuclear/caudate	R	15	−6	−12	4.65	5670	0.032

MNI: Montreal Neurological Institute; OFC: orbitofrontal cortex; IFG: inferior frontal gyrus; MFG: middle frontal gyrus; SOG: superior occipital gyrus; DLPFC: dorsolateral prefrontal cortex.

*P* values were family-wise error corrected for multiple comparisons with the nonisotropic adjusted cluster level [59] with an underlying voxel level of *P* < 0.001.

**Table 6:** White matter clusters in which tract-based spatial statistical analyses have shown significant increases in fractional anisotropy (FA) after training in Group C.

	Size (mm^3^)	MAX (1 − *P*)	MNI peak coordinates (mm)			MNI COG coordinates (mm)		
			*x*	*y*	*z*	*x*	*y*	*z*
L	1107	0.970	−23	−59	37	−19.5	−57.4	38.4
L	283	0.961	−28	−34	18	−29.1	−41.2	17.2
L	119	0.959	−42	−45	9	−39.3	−44.0	13.8
L	51	0.964	−22	−59	29	−23.1	−57.7	27.5
L	1	0.950	−32	−48	26	−32.0	−48.0	26.0
L	1	0.951	−36	−51	−5	−36.0	−51.0	−5.0

The listed clusters were defined by threshold-free cluster enhancement-corrected (1 − *P*) > 0.95.

COG: center of gravity.

## References

[1] Oxley J., Langford J., Charlton J. (2010). The safe mobility of older drivers: a challenge for urban road designers. *Journal of Transport Geography*.

[2] Inoue K., Fukunaga T., Okazaki Y., Fujita Y. (2012). Detailed discussion on evidence for the further prevention of traffic fatalities in Japan: a comparison of trends in three countries. *Medicine, Science and the Law*.

[3] Oxley J., Whelan M. (2008). It cannot be all about safety: the benefits of prolonged mobility. *Traffic Injury Prevention*.

[4] Anstey K. J., Wood J., Lord S., Walker J. G. (2005). Cognitive, sensory and physical factors enabling driving safety in older adults. *Clinical Psychology Review*.

[5] Dawson J. D., Anderson S. W., Uc E. Y., Dastrup E., Rizzo M. (2009). Predictors of driving safety in early Alzheimer disease. *Neurology*.

[6] Richardson E. D., Marottoli R. A. (2003). Visual attention and driving behaviors among community-living older persons. *Journals of Gerontology Series A: Biological Sciences and Medical Sciences*.

[7] Bédard M., Weaver B., Darzinš P., Porter M. M. (2008). Predicting driving performance in older adults: we are not there yet!. *Traffic Injury Prevention*.

[8] Ball K., Edwards J. D., Ross L. A. (2007). The impact of speed of processing training on cognitive and everyday functions. *Journals of Gerontology, Series B: Psychological Sciences and Social Sciences*.

[9] Edwards J. D., Delahunt P. B., Mahncke H. W. (2009). Cognitive speed of processing training delays driving cessation. *Journals of Gerontology Series A: Biological Sciences and Medical Sciences*.

[10] Edwards J. D., Myers C., Ross L. A. (2009). The longitudinal impact of cognitive speed of processing training on driving mobility. *Gerontologist*.

[11] Roenker D. L., Cissell G. M., Ball K. K., Wadley V. G., Edwards J. D. (2003). Speed-of-processing and driving simulator training result in improved driving performance. *Human Factors*.

[12] Romoser M. R. E., Fisher D. L. (2009). The effect of active versus passive training strategies on improving older drivers' scanning in intersections. *Human Factors*.

[13] Cassavaugh N. D., Kramer A. F. (2009). Transfer of computer-based training to simulated driving in older adults. *Applied Ergonomics*.

[14] Wolf D., Fischer F. U., Fesenbeckh J. (2014). Structural integrity of the corpus callosum predicts long-term transfer of fluid intelligence-related training gains in normal aging. *Human Brain Mapping*.

[15] Engvig A., Fjell A. M., Westlye L. T. (2012). Memory training impacts short-term changes in aging white matter: a longitudinal diffusion tensor imaging study. *Human Brain Mapping*.

[16] Erickson K. I., Voss M. W., Prakash R. S. (2011). Exercise training increases size of hippocampus and improves memory. *Proceedings of the National Academy of Sciences of the United States of America*.

[17] Engvig A., Fjell A. M., Westlye L. T. (2010). Effects of memory training on cortical thickness in the elderly. *NeuroImage*.

[18] Boyke J., Driemeyer J., Gaser C., Büchel C., May A. (2008). Training-induced brain structure changes in the elderly. *The Journal of Neuroscience*.

[19] Colcombe S. J., Erickson K. I., Scalf P. E. (2006). Aerobic exercise training increases brain volume in aging humans. *Journals of Gerontology—Series A Biological Sciences and Medical Sciences*.

[20] Pieramico V., Esposito R., Sensi F. (2012). Combination training in aging individuals modifies functional connectivity and cognition, and is potentially affected by dopamine-related genes. *PLoS ONE*.

[21] Mozolic J. L., Hayasaka S., Laurienti P. J. (2010). A cognitive training intervention increases resting cerebral blood flow in healthy older adults. *Frontiers in Human Neuroscience*.

[22] Owsley C., Ball K., McGwin G. (1998). Visual processing impairment and risk of motor vehicle crash among older adults. *The Journal of the American Medical Association*.

[23] Verghese J., LeValley A., Derby C. (2006). Leisure activities and the risk of amnestic mild cognitive impairment in the elderly. *Neurology*.

[24] Tranter L. J., Koutstaal W. (2008). Age and flexible thinking: an experimental demonstration of the beneficial effects of increased cognitively stimulating activity on fluid intelligence in healthy older adults. *Neuropsychology, Development, and Cognition, Section B: Aging and Cognition*.

[25] Akbaraly T. N., Portet F., Fustinoni S. (2009). Leisure activities and the risk of dementia in the elderly: results from the three-city study. *Neurology*.

[26] Verghese J., Lipton R. B., Katz M. J. (2003). Leisure activities and the risk of dementia in the elderly. *The New England Journal of Medicine*.

[27] Hambrick D. Z., Salthouse T. A., Meinz E. J. (1999). Predictors of crossword puzzle proficiency and moderators of age-cognition relations. *Journal of Experimental Psychology: General*.

[28] Salthouse T. A. (2006). Mental exercise and mental aging—evaluating the validity of the ‘use it or lose it’ hypothesis. *Perspectives on Psychological Science*.

[29] Schooler C. (2007). Use it—and keep it, longer, probably: a reply to salthouse. *Perspectives on Psychological Science*.

[30] Burciu R. G., Fritsche N., Granert O. (2013). Brain changes associated with postural training in patients with cerebellar degeneration: a voxel-based morphometry study. *Journal of Neuroscience*.

[31] Takeuchi H., Taki Y., Sassa Y. (2011). Working memory training using mental calculation impacts regional gray matter of the frontal and parietal regions. *PLoS ONE*.

[32] Bezzola L., Mérillat S., Gaser C., Jäncke L. (2011). Training-induced neural plasticity in golf novices. *Journal of Neuroscience*.

[33] Takeuchi H., Taki Y., Hashizume H. (2011). Effects of training of processing speed on neural systems. *Journal of Neuroscience*.

[34] Scholz J., Klein M. C., Behrens T. E. J., Johansen-Berg H. (2009). Training induces changes in white-matter architecture. *Nature Neuroscience*.

[35] Lee B., Park J.-Y., Jung W. H. (2010). White matter neuroplastic changes in long-term trained players of the game of ‘Baduk’ (GO): a voxel-based diffusion-tensor imaging study. *NeuroImage*.

[36] Lövdén M., Bodammer N. C., Kühn S. (2010). Experience-dependent plasticity of white-matter microstructure extends into old age. *Neuropsychologia*.

[37] Ziegler D. A., Piguet O., Salat D. H., Prince K., Connally E., Corkin S. (2010). Cognition in healthy aging is related to regional white matter integrity, but not cortical thickness. *Neurobiology of Aging*.

[38] Takeuchi H., Sekiguchi A., Taki Y. (2010). Training of working memory impacts structural connectivity. *Journal of Neuroscience*.

[39] Smith S. M., Jenkinson M., Johansen-Berg H. (2006). Tract-based spatial statistics: voxelwise analysis of multi-subject diffusion data. *NeuroImage*.

[40] Folstein M. F., Folstein S. E., McHugh P. R. (1975). ‘Mini-mental state’. A practical method for grading the cognitive state of patients for the clinician. *Journal of Psychiatric Research*.

[41] Classen S., Wang Y., Crizzle A. M., Winter S. M., Lanford D. N. (2013). Gender differences among older drivers in a comprehensive driving evaluation. *Accident Analysis and Prevention*.

[42] Moher D., Hopewell S., Schulz K. F. (2012). CONSORT 2010 explanation and elaboration: updated guidelines for reporting parallel group randomised trials. *International Journal of Surgery*.

[43] Wechsler D. (1997). *Wechsler Adult Intelligence Scale*.

[44] Dubois B., Slachevsky A., Litvan I., Pillon B. (2000). The FAB: a frontal assessment battery at bedside. *Neurology*.

[45] Lezak M. (1995). *Neuropsychological Assessment*.

[46] Reitan R. M. (1958). Validity of the trail making test as an indicator of organic brain damage. *Perceptual and Motor Skills*.

[47] Smith A. (1984). *Symbol Digit Modalities Test Manual—Revised*.

[48] Wechsler D. (1987). *Wechsler Memory Scale*.

[49] Benton A. (1963). *The Revised Visual Retention Test: Clinical and Experimental Applications*.

[50] Shin M.-S., Park S.-Y., Park S.-R., Seol S.-H., Kwon J. S. (2006). Clinical and empirical applications of the Rey-Osterrieth complex figure test. *Nature Protocols*.

[51] Schmidt M. (1996). *Rey Auditory Verbal Learning Test: RAVLT: A Handbook*.

[52] Benton A., Hamsher K., Varney N., Spreen O. (1983). *Judgement of Line Orientation*.

[53] Rizzo M., McGehee D. V., Dawson J. D., Anderson S. N. (2001). Simulated car crashes at intersections in drivers with Alzheimer disease. *Alzheimer Disease and Associated Disorders*.

[54] Schmiedek F., Lövdén M., Lindenberger U. (2010). Hundred days of cognitive training enhance broad cognitive abilities in adulthood: findings from the COGITO study. *Frontiers in Aging Neuroscience*.

[55] Uc E. Y., Rizzo M., Anderson S. W., Shi Q., Dawson J. D. (2005). Driver landmark and traffic sign identification in early Alzheimer's disease. *Journal of Neurology, Neurosurgery and Psychiatry*.

[56] Benjamini Y., Hochberg Y. (1995). Controlling the false discovery rate: a practical and powerful approach to multiple testing. *Journal of the Royal Statistical Society. Series B. Methodological*.

[57] Ashburner J. (2007). A fast diffeomorphic image registration algorithm. *NeuroImage*.

[58] Good C. D., Johnsrude I. S., Ashburner J., Henson R. N. A., Friston K. J., Frackowiak R. S. J. (2001). A voxel-based morphometric study of ageing in 465 normal adult human brains. *NeuroImage*.

[59] Hayasaka S., Phan K. L., Liberzon I., Worsley K. J., Nichols T. E. (2004). Nonstationary cluster-size inference with random field and permutation methods. *NeuroImage*.

[60] Nichols T. E., Holmes A. P. (2002). Nonparametric permutation tests for functional neuroimaging: a primer with examples. *Human Brain Mapping*.

[61] Smith S. M., Nichols T. E. (2009). Threshold-free cluster enhancement: addressing problems of smoothing, threshold dependence and localisation in cluster inference. *NeuroImage*.

[62] Takei Scientific Instruments

[63] The Traffic Division of the Scientific Police Institute *National Police Agency System Guidelines*.

[64] Erickson K. I., Kramer A. F. (2009). Aerobic exercise effects on cognitive and neural plasticity in older adults. *British Journal of Sports Medicine*.

[65] Scherder E. J. A., van Paasschen J., Deijen J.-B. (2005). Physical activity and executive functions in the elderly with mild cognitive impairment. *Aging and Mental Health*.

[66] Kringelbach M. L. (2005). The human orbitofrontal cortex: linking reward to hedonic experience. *Nature Reviews Neuroscience*.

[67] Matsuo K., Nicoletti M., Nemoto K. (2009). A voxel-based morphometry study of frontal gray matter correlates of impulsivity. *Human Brain Mapping*.

[68] Chee M. W. L., Chen K. H. M., Zheng H. (2009). Cognitive function and brain structure correlations in healthy elderly East Asians. *NeuroImage*.

[69] Corbetta M., Patel G., Shulman G. L. (2008). The reorienting system of the human brain: from environment to theory of mind. *Neuron*.

[70] Barbey A. K., Koenigs M., Grafman J. (2013). Dorsolateral prefrontal contributions to human working memory. *Cortex*.

[71] D'Esposito M., Postle B. R., Rypma B. (2000). Prefrontal cortical contributions to working memory: evidence from event-related fMRI studies. *Experimental Brain Research*.

[72] Olesen P. J., Westerberg H., Klingberg T. (2004). Increased prefrontal and parietal activity after training of working memory. *Nature Neuroscience*.

[73] Turken A., Whitfield-Gabrieli S., Bammer R., Baldo J. V., Dronkers N. F., Gabrieli J. D. E. (2008). Cognitive processing speed and the structure of white matter pathways: convergent evidence from normal variation and lesion studies. *NeuroImage*.

[74] Owen A. M., McMillan K. M., Laird A. R., Bullmore E. (2005). N-back working memory paradigm: a meta-analysis of normative functional neuroimaging studies. *Human Brain Mapping*.

[75] Wallentin M., Roepstorff A., Glover R., Burgess N. (2006). Parallel memory systems for talking about location and age in precuneus, caudate and Broca's region. *NeuroImage*.

[76] Lewis S. J. G., Dove A., Bobbins T. W., Barker R. A., Owen A. M. (2004). Striatal contributions to working memory: a functional magnetic resonance imaging study in humans. *European Journal of Neuroscience*.

[77] Kanai R., Rees G. (2011). The structural basis of inter-individual differences in human behaviour and cognition. *Nature Reviews Neuroscience*.

[78] Raz N., Lindenberger U., Rodrigue K. M. (2005). Regional brain changes in aging healthy adults: general trends, individual differences and modifiers. *Cerebral Cortex*.

[79] Fjell A. M., Westlye L. T., Amlien I. (2009). High consistency of regional cortical thinning in aging across multiple samples. *Cerebral Cortex*.

[80] Edwards J. D., Wadley V. G., Myers R. S., Roenker D. L., Cissell G. M., Ball K. K. (2002). Transfer of a speed of processing intervention to near and far cognitive functions. *Gerontology*.

[81] Li S.-C., Schmiedek F., Huxhold O., Röcke C., Smith J., Lindenberger U. (2008). Working memory plasticity in old age: practice gain, transfer, and maintenance. *Psychology and Aging*.

[82] Dahlin E., Nyberg L., Bäckman L., Neely A. S. (2008). Plasticity of executive functioning in young and older adults: immediate training gains, transfer, and long-term maintenance. *Psychology and Aging*.

[83] Borella E., Carretti B., Riboldi F., De Beni R. (2010). Working memory training in older adults: evidence of transfer and maintenance effects. *Psychology and Aging*.

[84] Nouchi R., Taki Y., Takeuchi H. (2012). Brain training game improves executive functions and processing speed in the elderly: a randomized controlled trial. *PLoS ONE*.

[85] Brehmer Y., Westerberg H., Bäckman L. (2012). Working-memory training in younger and older adults: training gains, transfer, and maintenance. *Frontiers in Human Neuroscience*.

[86] Sakai H., Takahara M., Honjo N. F., Doi S., Sadato N., Uchiyama Y. (2012). Regional frontal gray matter volume associated with executive function capacity as a risk factor for vehicle crashes in normal aging adults. *PLoS ONE*.

[87] Ball K., Berch D. B., Helmers K. F. (2002). Effects of cognitive training interventions with older adults: a randomized controlled trial. *The Journal of the American Medical Association*.

[88] Willis S. L., Tennstedt S. L., Marsiske M. (2006). Long-term effects of cognitive training on everyday functional outcomes in older adults. *The Journal of the American Medical Association*.

[89] Lustig C., Shah P., Seidler R., Reuter-Lorenz P. A. (2009). Aging, training, and the brain: a review and future directions. *Neuropsychology Review*.

