# Supplementary material for: Effects of Different Types of Cognitive Training on Cognitive Function, Brain Structure, and Driving Safety in Senior Daily Drivers: A Pilot Study
Source: Behav Neurol. 2015 Jun 16;2015:525901. doi: 10.1155/2015/525901 (PMC4487932; doi:10.1155/2015/525901)
Supplement: Supplementary file 1 — Supplementary Materials provide additional information and data on the cognitive training tasks and tests. [file 525901.f1.pdf]

Supplementary Figures

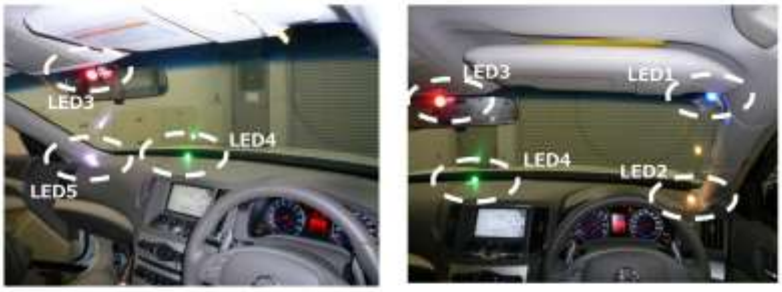

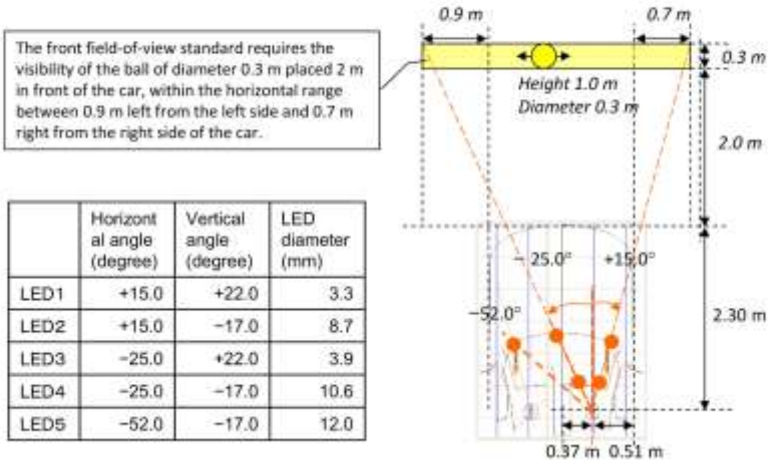

	Horizontal angle (degree)	Vertical angle (degree)	LED diameter (mm)
LED1	+15.0	+22.0	3.3
LED2	+15.0	−17.0	8.7
LED3	−25.0	+22.0	3.9
LED4	−25.0	−17.0	10.6
LED5	−52.0	−17.0	12.0

Figure S1 Configuration of the light-emitting diode lights of the onboard cognitive training system. Position of the four LEDs (LED1: top-right, LED2: bottom-right, LED3: top-left, and LED4: bottom-left) were determined in accordance with the front field-of-view standard established by law. Their horizontal angles from the driver's eye position (EP) were on the border of the horizontal field defined by the standard (yellow area). Their vertical positions were on the border of the front window view, ensuring the vertical sight defined by the standard. The fifth LED was placed beside the distant side mirror, considering the importance of keeping attention on it for driving safety. LED diameters were adjusted by masking so that the perceived sizes of the light stimuli are the same.

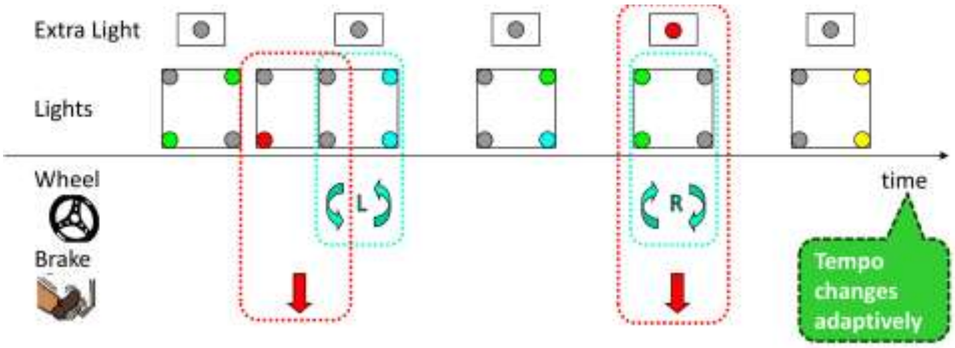

Figure S2 Illustration of the rules of the immediate response task

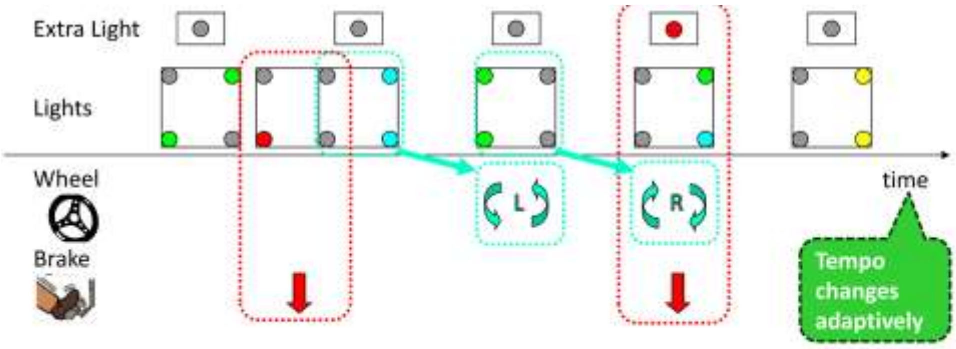

Figure S3 Illustration of the rules of the delayed response task. This example describes the case in which the delay that was required for the responses to the periodic stimuli was set to n = 1.

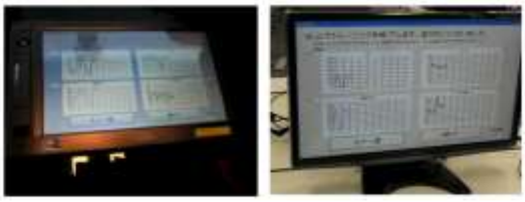

Figure S4 Performance feedback of the cognitive training systems. Left: The onboard training system (Group V); right: the training system on a personal computer (Group P).

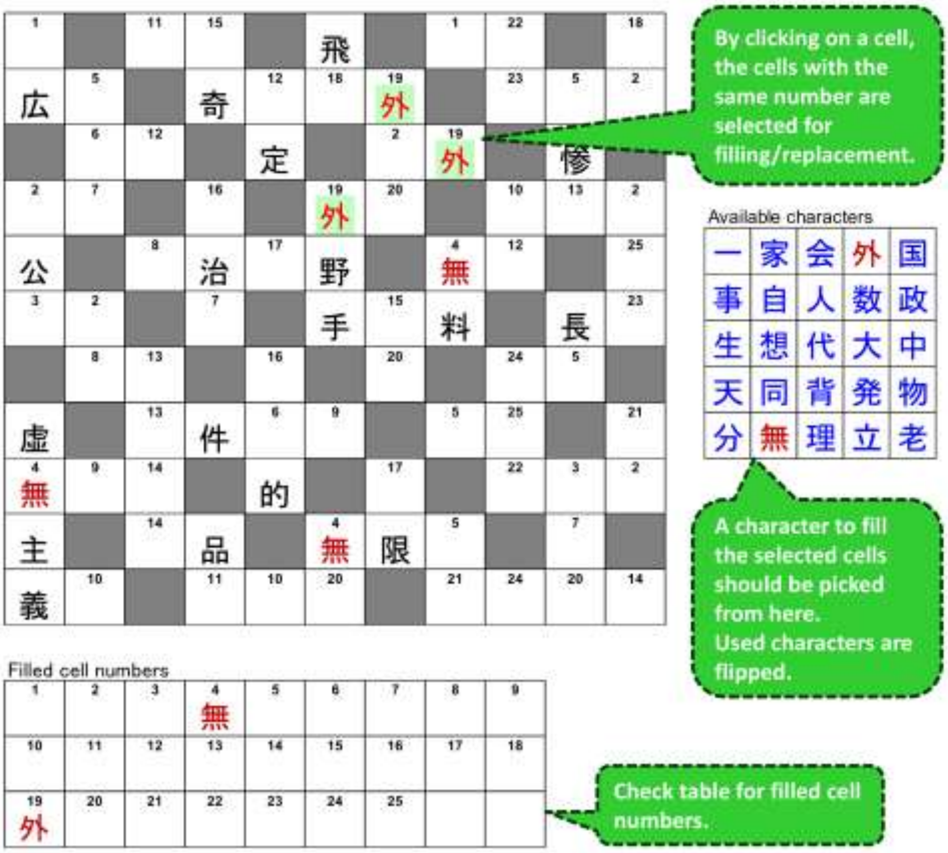

Figure S5 Example problem of the number crossword puzzle, which was the training task for Group C.

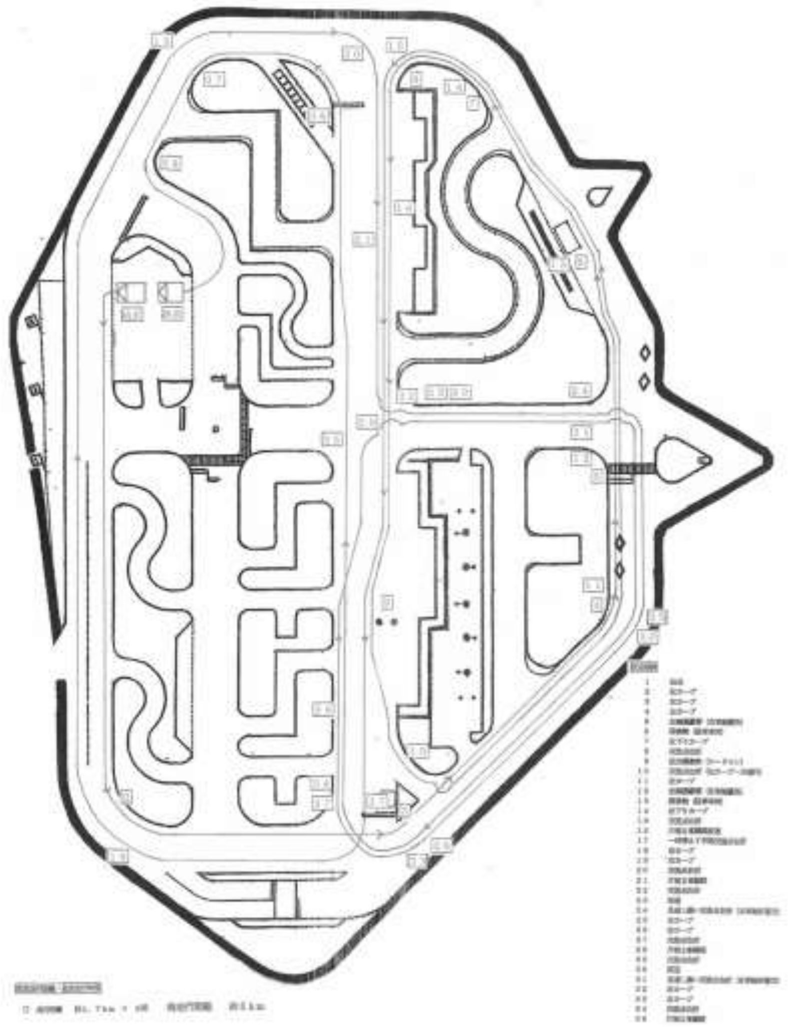

Figure S6 Course used in the on-road driving safety evaluation.

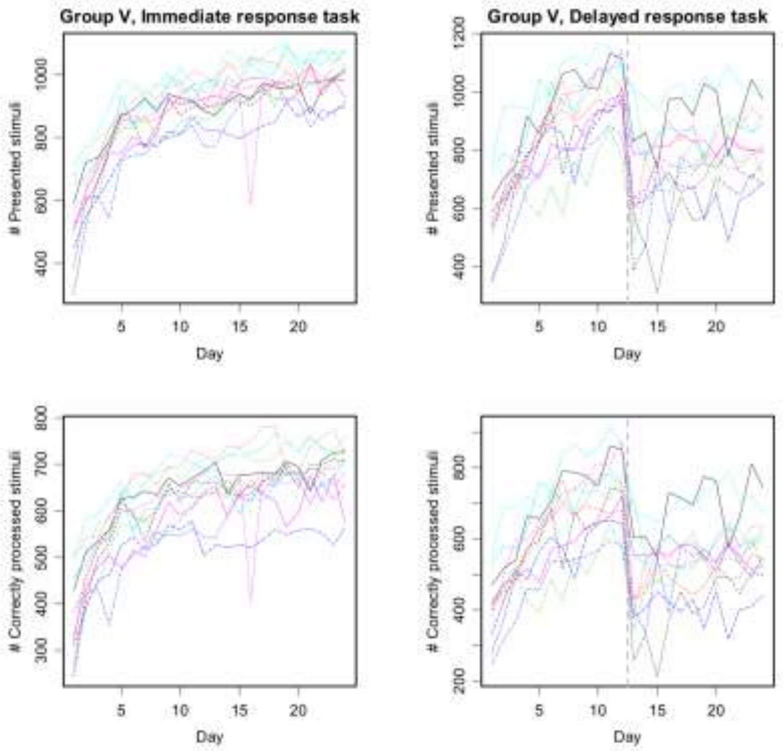

Figure S7 Performance changes in the two training tasks over the 24 training visits for the 12 subjects in Group V. Upper left: Number of stimuli presented in the immediate response task. Lower left: Number of stimuli correctly processed in the immediate response task. Upper right: Number of stimuli presented in the delayed response task. Lower right: Number of stimuli correctly processed in the delayed response task. For each task, all stimuli counts were summed over the two sessions on each training visit. The central vertical dotted lines in the graphs on the right indicate the switch of required delay from n = 1 to 2 (see Text).

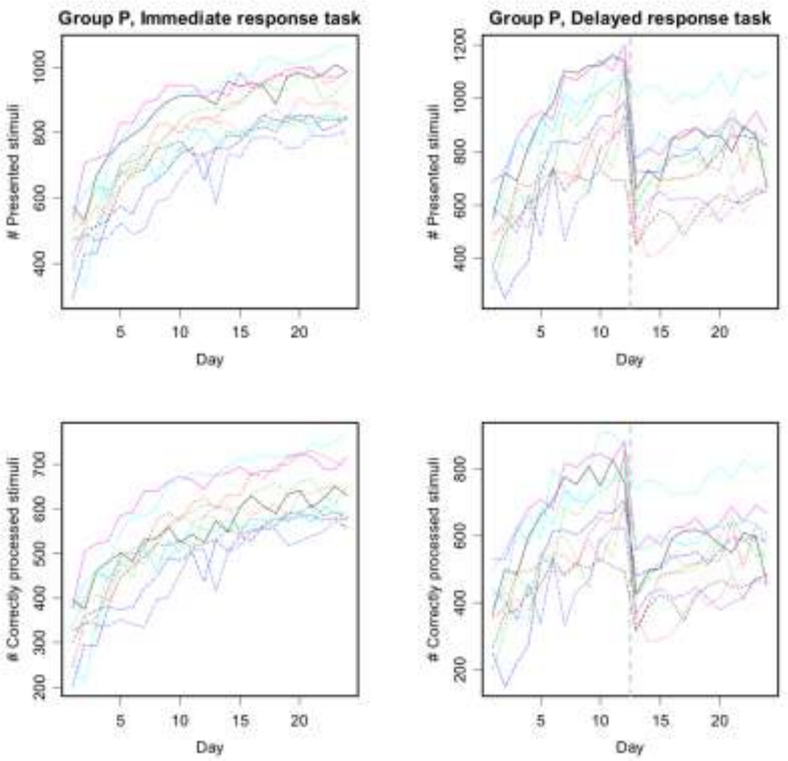

Figure S8 Performance change of the two training tasks over the 24 training visits for the 11 subjects in Group P. Upper left: Number of stimuli presented in the immediate response task. Lower left: Number of stimuli correctly processed in the immediate response task. Upper right: Number of stimuli presented in the delayed response task. Lower right: Number of stimuli correctly processed in the delayed response task. For each task, all stimuli counts were summed over the two sessions on each training visit. The central vertical dotted lines in the graphs on the right indicate the switch in the required delay from n = 1 to 2 (see Text).
